# Synthesis and *in-vitro* anticancer evaluation of polyarsenicals related to the marine sponge derived Arsenicin A

**DOI:** 10.1038/s41598-017-11566-6

**Published:** 2017-09-14

**Authors:** Ines Mancini, Matteo Planchestainer, Andrea Defant

**Affiliations:** 1 Università degli studi di Trento, Dipartimento di Fisica, Laboratorio di Chimica Bioorganica, via Sommarive 14, 38123 Povo, Trento Italy; 2 School of Chemistry, University of Nottingham, University Park, Nottingham, NG72RD UK

## Abstract

In the light of the promising bioactivity of the tetraarsenic marine metabolite arsenicin A, the dimethyl analogue **2** and four isomeric methylene homologues (including the natural product itself) were obtained using a one-pot microwave-assisted synthesis, starting from arsenic (III) oxide. Due to the poor diagnostic value of the NMR technique in the structural elucidation of these molecules, they were fully characterized by mass spectrometry and infrared (IR)-spectroscopy, comparing density functional theory (DFT) simulated and experimental spectra. This synthetic procedure provided a fast and efficient access to the cytotoxicity evaluation of organoarsenical leads of the natural hit molecule. From *in vitro* screening, each tested compound resulted in being more active than the FDA-approved arsenic trioxide, with the most lipophilic molecule in the series showing the best growth inhibition of both leukemia and solid tumor cell lines. These results may open promising perspectives in the development of new more potent and selective arsenical drugs against solid tumors.

## Introduction

Arsenic is a paradoxical element, on one hand it is a highly toxic and a notorious carcinogen while on the other it can be a charming medicine. Salvarsan for example was the first arsenic based drug, synthesized for the effective treatment of the infectious disease syphilis and sleeping sickness^1^. Arsenic oxides, extensively exploited in traditional Chinese medicine, have also been investigated as novel chemotherapeutic compounds^2^. In 2000, arsenic trioxide (ATO) was approved by the Food and Drug Administration (FDA) as a chemotherapeutic agent and at present it is one of the most effective drugs in the treatment of acute promyelocytic leukemia (APL), leading to complete remission in a high percentage of patients^3, 4^. Similar activity was also seen for arsenolite (As_4_O_6_), which showed apoptosis-inducing effects against human leukemic and some solid tumor cells^2^.

These positive results prompted significant interest in the use of arsenic oxides for the treatment of solid tumors. While the use of ATO as a single agent showed little benefit, remarkable clinical outcomes were reported when used in combination with other chemotherapeutic agents. A comparative study showed that As_4_O_6_ is a better inhibitor of human cervical cancer than As_2_O_3_^3, 5^. It has also been demonstrated that arsenic tetroxide has a greater potential against human cervix, gastric, and head cancer cells when used in combination^6^, e.g. with paclitaxel^7^. Despite the success of arsenic trioxide in the treatment of APL, its efficacy towards solid tumors is limited by its poor pharmacokinetics and dose-limiting toxicity. Nanotechnology however offers an attractive solution to these shorthcomings^8^. For example, porous silica smart nanoparticles have been developed as nanocarries for arsenic trioxide exhibiting much higher cytotoxicity to a variety of cancer cells than the free compound^9^.

**Figure 1:**
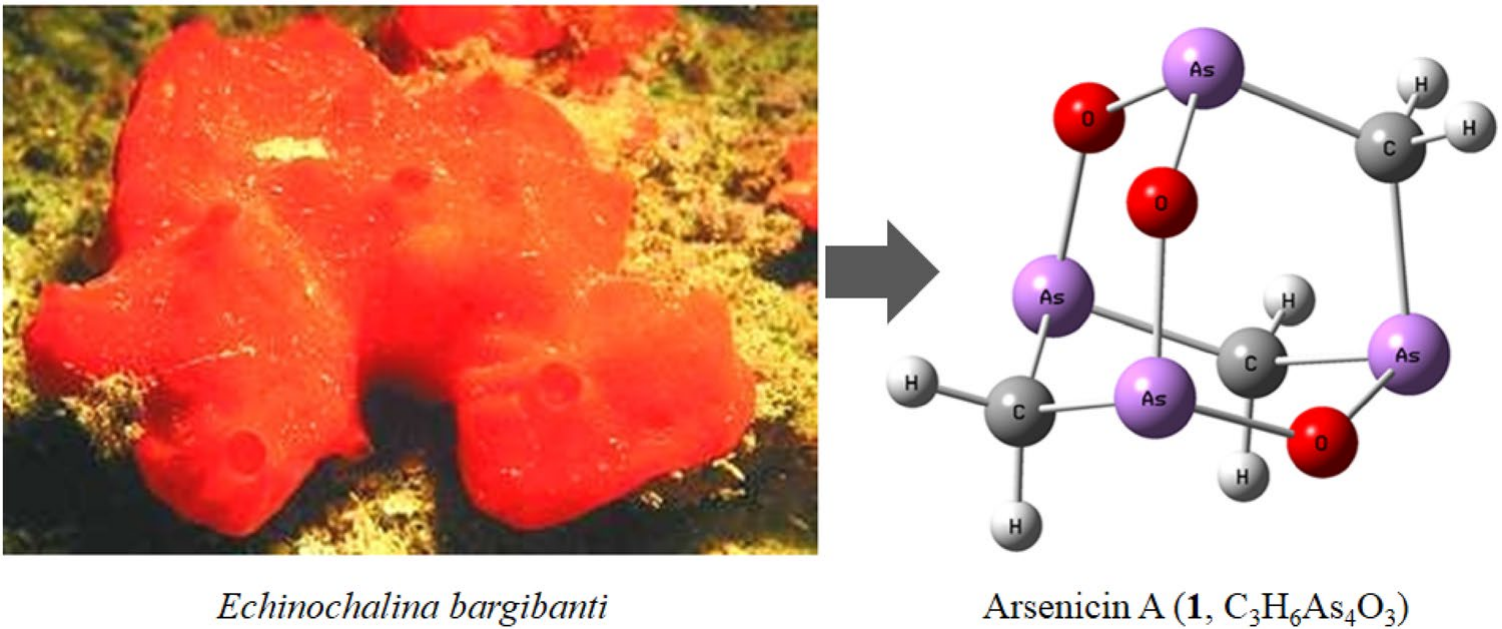
Arsenicin A. Molecular structure of arsenicin A (**1**), isolated from the New Caledonian sponge *Echinochalina bargibanti*.

Historically, natural products have played a prominent relevant role in medicine and continue to do so today. Marine metabolites have gained significant attention as interesting leads, employing their peculiar molecular structures and their natural role in protecting the source organism. In this framework, the marine product arsenicin A (=1,2,4,6-trioxa-1,3,5,7-tetrarsa-tricyclo [3.3.1.13,7] decane), isolated in a small amount from the phloecilosclerid sponge *Echinochalina bargibanti* collected along the New Caledonian coasts, is an example. Reported as the first organic polyarsenic compound ever found naturally, its adamantane cage resembles arsenolite structure (Fig. 1)^10^. It is unusual, because natural organoarsenicals isolated so far included monoarsenic metabolites, mostly as apolar methylated forms, or water soluble betaine and arsenosugars^11^.

Recently, racemic arsenicin A was synthesized in five steps and 36% overall yield starting from phenylarsinic acid and its crystal structure was described. In a cytotoxicity evaluation on some human carcinoma cell lines^12, 13^, it proved to be very efficient in inducing cell death in acute promyelocytic leukemia cell lines and in stopping the advancement of pancreatic adenocarcinoma and glioblastoma^13^.

Based on knowledge acquired so far, the potential of arsenicin A as a promising lead in drug development warrants structure-activity relationship (SAR) studies on synthetic analogues. The purpose of this work is to broaden the molecular diversity by related compounds, accessible by an efficient synthetic method and showing a more potent bioactivity.

Here we report a fast microwave assisted method for the synthesis of a series of tetra-arsenic compounds (**1–5**) including racemic arsenicin A and their cytotoxicity evaluation on a wide panel of human cancer cells.

## Results and Discussion

### Synthesis and structure elucidation

**Figure 2:**
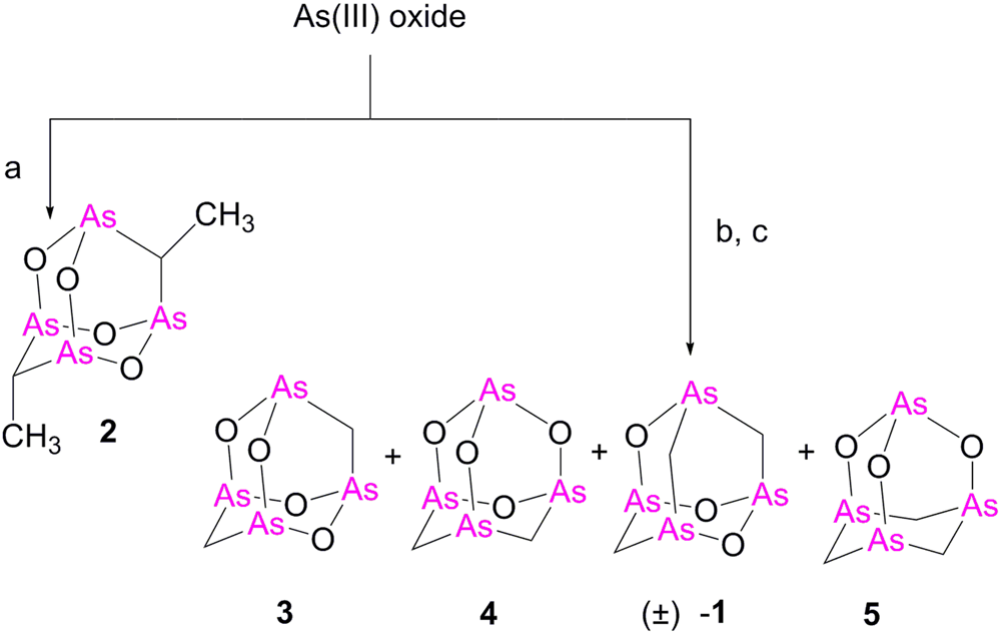
Synthesis of arsenicals. Reaction conditions: (**a**) CH_3_CH_2_COOH, (CH_3_CH_2_CO)_2_O, K_2_CO_3_ MW,160 °C, 20 min; addition of H_2_O, MW 80 °C, 20 min, 82%; (**b**) CH_3_COOH, (CH_3_CO)_2_O, K_2_CO_3_ MW, 138 °C, 30 min; addition of H_2_O, MW 80 °C, 30 min, 38%; (**c**) HPLC separation.

The synthesis of an analogue was crucial for the challenging structural determination of the natural metabolite Arsenicin A^10^. The model compound 9,10-dimethyl-2,4,6,8-tetraoxa-1,3,5,7,-tetraarsa-tricyclo[3.3.1.13,7]decane (**2**, Fig. 2) was obtained by heating at 160 °C, As_2_O_3_, K_2_CO_3_ and propionic acid in propionic anhydride according to procedure previously outlined by Keppler and coworkers, who confirmed the structure by X-ray diffraction analysis^14^.

This one-pot method applied to the proper carboxylic acid/anhydride pair was recognized as an attractive procedure for the synthesis of a series of arsenicin A-like molecules. The replacement of conventional heating by microwave (MW) irradiation, has significantly reduced the reaction time required to produce **2** ^10, 14^. In traditional method, a two-hour heating of the reactants is followed by a hydrolysis step which requires an additional hour. The use of microwave irradiation has efficiently reduced these lengthy steps to 20 minutes each under the same temperature conditions. HPLC analysis was applied to determinate the selectivity in the production of the compound **2**. The crude reaction mixture indeed showed a major peak at 6.44 min (CN stationary phase, hexane, flow 5 mL min^−1^, UV detection at 254 nm and 240 nm) with two considerably lower peaks.

2,4,6,8-Tetraoxa-1,3,5,7-tetraarsa-adamantane (**3**)^15^, the analogue of molecular composition C_2_H_4_As_4_O_4_, was expected from the MW-assisted reaction of arsenic (III) oxide with K_2_CO_3_, acetic acid and acetic anhydride (1:1:4:8 molar equivalent, respectively) under optimized conditions (138 °C for 0.5 h, followed by hydrolysis at 80 °C for 0.5 h). The atmospheric pressure chemical ionization (APCI)-MS spectrum in positive ion mode of the crude reaction mixture, revealed a signal at *m/z* 392.7 attributable to compound **3**. However, an additional signal at *m/z* 390.7 was observed, indicative of product(s) with the molecular composition C_3_H_6_As_4_O_3_. Further purification by HPLC technique (CN stationary phase, hexane/AcOEt 96:4, flow 5 mL•min^−1^, λ = 254 nm) provided the isomeric product pairs **3** and **4**, and the C_3_H_6_As_4_O_3_ isomers arsenicin A (**1**) and **5** (Fig. 2). These products are listed in order of their higher retention times, which correspond to the increased polarity of progressively methylene-substituted homologues.

It is interesting to note that the UV detection value utilized in the HPLC purification of these arsenical compounds was based on the behaviour of arsenicin A. Even in the absence of an obvious chromophore, arsenicin A is absorbent at relatively high values of UV wavelength. This feature was supported by time-dependent density functional theory (TD-DFT) calculations^16^. Ultraviolet spectra recorded on purified arsenicals **3** and **5** showed a similar response with intense absorption bands at 230/276 nm and 230/284 nm, respectively.

The higher selectivity in the production of **2** with respect to the analogue **3** could be attributed to the greater steric hindrance of a methyl substituent on the adamantane cage.

**Figure 3:**
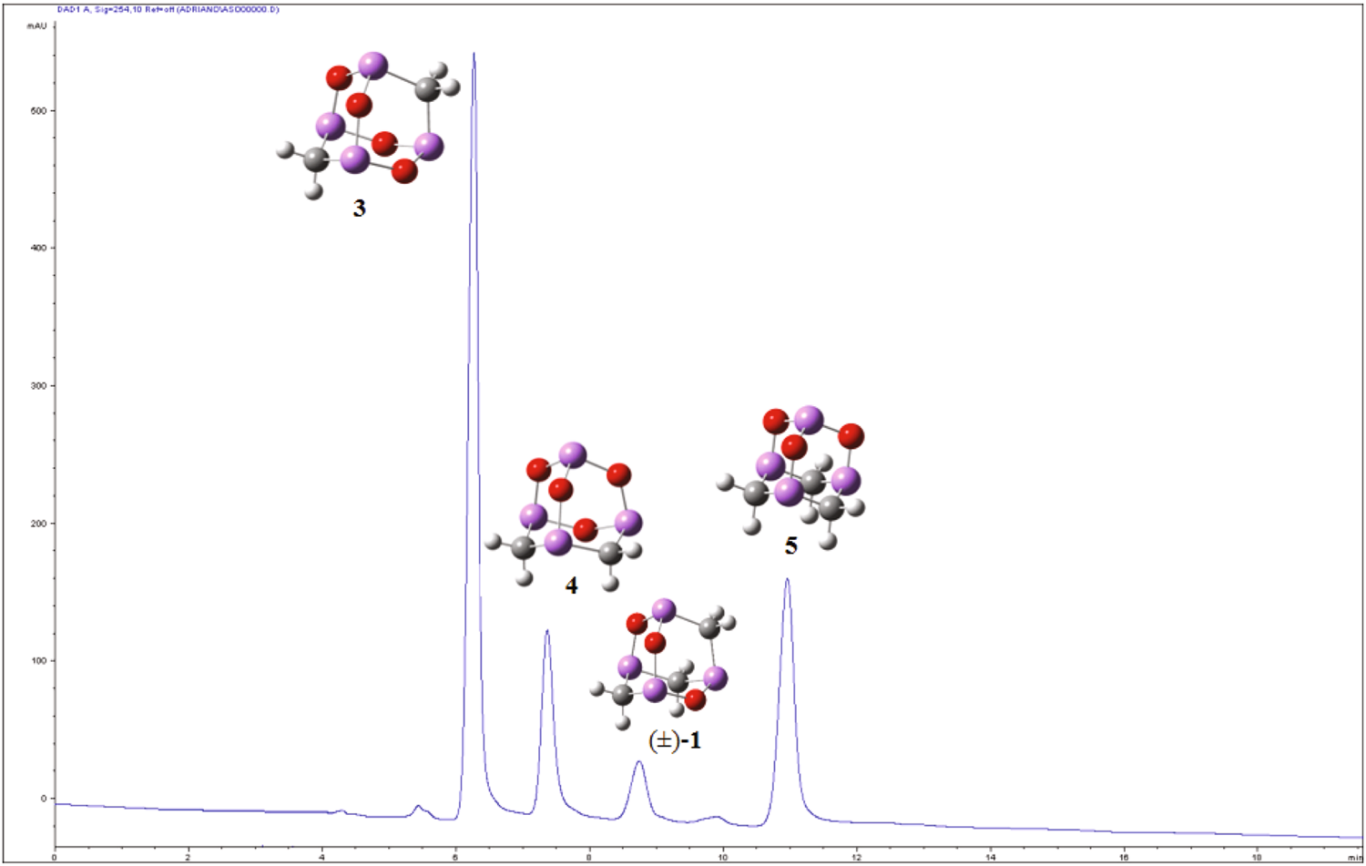
Preparative HPLC–DAD analysis. Chromatogram of the crude mixture from MW-assisted reaction of arsenic (III) oxide with K_2_CO_3_/acetic acid/acetic anhydride (LiChrospher CN column, hexane/AcOEt 96:4, flow 5 mLmin^−1^, 254 nm) Compounds **3** (t_R_ = 6.2 min), 4 (t_R_ = 7.4 min), (±)-arsenicin A (**1**, t_R_ = 8.7 min) and 5 (t_R_ = 11.0 min).

The most abundant product **3** was obtained as a whitish powder after evaporation of the solution eluted at 6.2 min by preparative HPLC analysis (CN column, hexane/AcOEt 96:4, λ = 245 nm), according the chromatographic profile reported in Fig. 3.

High resolution electron ionization (HREI)-MS analysis of purified compound **3** provided a signal at *m/z* 391.6972 (C_2_H_4_As_4_O_4_), and a fragment ion (*m/z* 361.6865, CH_2_As_4_O_3_) through the loss of a formaldehyde molecule, as for the natural arsenicin A^10^. ^1^H NMR spectrum in CDCl_3_ showed a lone singlet at δ_H_ 1.85, which correlated to δ_C_ 30.60 by the HSQC experiment. This pinpointed the proposed symmetrical structure of the molecule, which was confirmed by favourable agreement between experimental and DFT-calculated IR frequencies, relying on the proven vibrational analysis as efficient tool in the structure elucidation of these polyarsenicals^10, 17^. In fact, the intense bands observed at 713 cm^−1^and 808 cm^−1^ and the weak band at 1076 cm^−1^ may be the result of As-C stretching, As-O stretching and C-H bending respectively, in comparison with the corresponding simulated values at 708, 780 and 1106 cm^−1^. All data complied with the methylenebis(arsaneoxide) structure reported by Betz *et al*.^18^, as a product by treating with aqueous ammonia the methylenebis (dichloroarsane) previously obtained from the reaction of arsenic trioxide with aluminium trichloride/acetyl chloride/thionyl chloride.

The new compound **4**, collected from the fraction eluted at 7.4 min by the same preparative HPLC purification of the reaction mixture (Fig. 3), displayed identical molecular composition to that of compound **3**. This was verified by high resolution EIMS (C_2_H_4_As_4_O_4_, *m/z* 391.6969), and the presence of an intense signal due to the loss of a formaldehyde molecule. Only two doublets at δ_H_ 2.71 and δ_H_ 1.71, in 1:1 ratio, were detected in the ^1^HNMR spectrum. The signals showed the same geminal constant coupling (*J* 13.8 Hz) and they were both connected to the same δ_C_ 23.71, because of the HSQC correlation. Due to structural restrictions, these signals were attributed to each of the two magnetically equivalent protons on two AsCH_2_As groups, symmetrically placed on the same adamantane six-membered heterocycle. This assignment is confirmed by excellent correlation in relative intensities and wavelength values (1156 cm^−1^ for C-H bending, 805 cm^−1^ for As-O stretching, 760 cm^−1^ for As-C stretching and 678 cm^−1^ for ring deformation) with the experimental FT-ATR-IR spectrum (1103, 796, 754 and 681 cm^−1^, respectively).

The HPLC peak eluted at 8.7 min corresponded to racemic form of natural arsenicin A (**1**). This was confirmed by the comparison of MS and NMR data with the ones reported for the natural metabolite^10^.

The most polar arsenical in the series was **5**, eluted at 11.0 min in the chromatogram of the preparative HPLC purification (Fig. 3). It was found to be an isomeric arsenicin A, supported by the same molecular formula as deduced by high resolution MS data (*m/z* 389.7175, C_3_H_6_As_4_O_3_) and by the loss of a formaldehyde molecule, a distinctive feature for this class of arsenicals. The two doublets in 1:1 ratio detected in the ^1^HNMR spectrum at δ_H_ 3.11 and δ_H_ 1.53 showed the same geminal coupling constant (*J* 13.8 Hz). These can be assigned to each of the pairs formed by three magnetically equivalent protons on three methylene groups. Through the HSQC experiments, both doublets showed correlation with the same δ_C_ 17.82, attributable to the three equivalent methylene carbon atoms. These ^1^HNMR values were in line with chemical shifts previously calculated at relativistic BP86-ZSO/TZ2P level for the optimized geometry at the B3LYP/6–311 G(2d,2p) level of theory. In detail, δ_H_ 2.99 and δ_H_ 1.33 were obtained for equivalent protons in equatorial and axial positions, respectively in the structure **5**, belonging to the symmetry group C3v^19^. In fact, molecule **5** was previously considered as a trial structure in the computational studies of both vibrational^10, 17^ and NMR^19^ spectroscopic analyses of arsenicals related to arsenicin A, but thus far this arsenical has never been synthesized. Compound **5** had been reported as the most stable structure in the evaluation of a 12-membered series of C_3_H_6_As_4_O_3_ isomeric arsenicals, owing to the high thermodynamic stability of the adamantane cage with three six-membered rings arranged in rigid chair configurations^19^. Moreover, in polyheteroadamantanes the dominant role of electrostatic effects in the stabilization has been evaluated for O and As derivatives^20^.

Also in this case, the molecular structure was endorsed by the comparison of experimental and calculated IR spectra. Most notably, the latter was previously reported as one of the virtual model compounds considered in the structural elucidation of the natural metabolite^10^. A good agreement was observed between the very strong absorption at 684 cm^−1^ and the calculated value at 670 cm^−1^ assigned to As-C stretching, the very strong band at 786 cm^−1^ and the value at 777 cm^−1^ assigned to As-O stretching, as well as the weak band at 1118 cm^−1^ and the value at 1162 cm^−1^, assigned to C-H bending.

The selectivity in the formation of these arsenicals was then investigated under certain experimental conditions. The relative amounts of the products were evaluated by comparing MW irradiation (138 °C, 1 h) and oil bath heating (140 °C, 5 h) conditions. It was established that products **3**, **4**, (±)-**1** and **5** were obtained in 71:7:1:21 ratio respectively by conventional heating and 82:10:3:5 ratio by MW irradiation. These values were deduced by integrating the area of product signals in ^1^HNMR spectra of the corresponding crude reaction mixtures. The results obtained indicated that MW-assisted procedure was a more selective towards the expected compound **3**, whereas the traditional method is the preferred choice when compound **5** is of interest.

To look deeper into the intriguing mechanism of this reaction, we verified that the reaction did not occur without K_2_CO_3_ or even in the absence of acetic anhydride. Whereas, the reaction proceeded quite well without the acetic acid, or when CH_3_COOH/K_2_CO_3_ was replaced with sodium acetate. This is reminiscent of the mechanism involved in the formation of cacodyl (=tetramethyldiarsine) and cacodyl oxide, which are components of Cadet’s fuming liquid^21^.

### Biological evaluation

**Figure 4:**
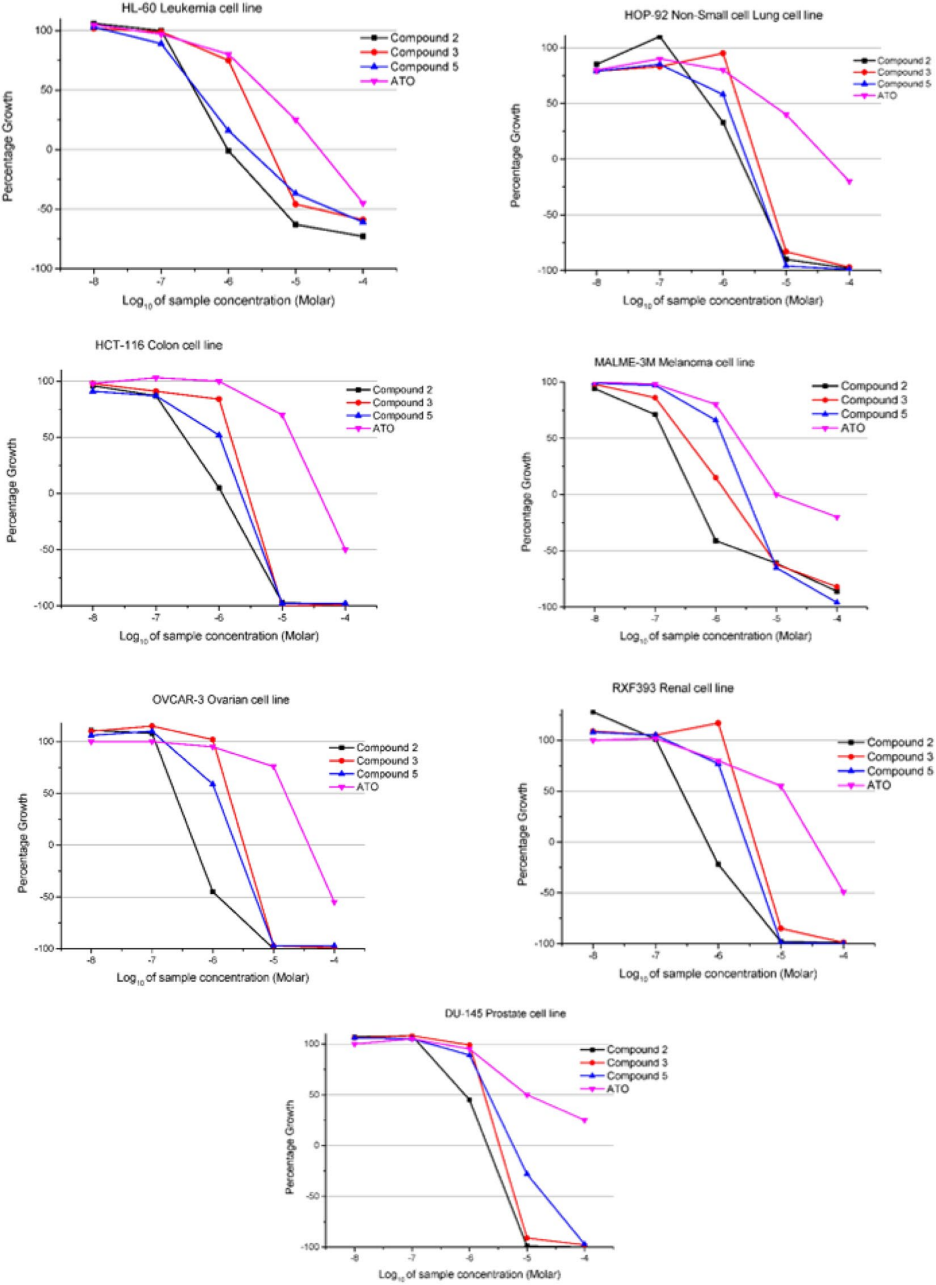
Biological tests. Dose response curves at NCI fixed protocol, (μM) for the indicated human cancer cell lines after treatment with compounds **2**, **3** and **5**, in comparison with arsenic trioxide (ATO). The curves are obtained at five concentrations (Log dilutions from 10^–4^ to 10^–8^ M). The concentration of each compound which inhibits the 50% of cell growth (GI_50_) can be deduced and are the values reported in Table 1.

Arsenical **2** and members of each different homologue series, compounds **3** and **5**, were accepted by the National Cancer Institute (NCI-USA) for *in vitro* cytotoxicity screening on their full panel of human cancer cell lines. The screening was based on an automated sulphorhodamine blue (SRB) cytotoxicity assay^22^. The most significant GI_50_ values are reported in Table 1, and compared to corresponding data for ATO. The average values of GI_50_ (MGM) obtained across the cell lines tested were lower than ATO, indicating a significant better activity for compound **2** in all the cases. In detail, it exhibited the most potent effect at a sub-micromolar concentration on the selected cell lines shown in the dose response curves reported in Fig. 4. A negligible difference was observed in the behaviour of arsenicals **3** and **5**. It is notable that inhibition effects are in the order **2** > **5** > **3** > ATO, in line with the increased number of C atoms in their corresponding molecular structures. Furthermore, the highest lipophilicity of **2** associated with a better membrane permeation may explain its highest activity against leukemia cells growth, as well as its very good cytotoxicity on solid tumor cells (Table 1, Fig. 4). The highest absolute activity was observed when arsenical **2** was tested on MALME-3M melanoma cells (GI_50_ = 0.176 µM). The best comparative effect was also obtained for compound **2** on colon HCT-116 line (GI_50_ = 0.299 µM). In this case, the concentration required to achieve 50% growth inhibition was 27-fold lower than that required for ATO.

**Table 1:** Inhibition of *in vitro* human cancer cell lines by compounds 2, 3, 5 and arsenic trioxide (ATO) as a reference (NCI available data, compd. 92859)

Cytotoxicity GI_50_ (μM)				
Cell lines	Compound 2	Compound 3	Compound 5	ATO
**Leukemia**				
CCRF-CEM	0.182 ± 0.028	1.25	0.307	0.501
HL-60(TB)	0.310 ± 0.107	1.60	0.339	2.512
K-562	0.468 ± 0.088	2.02	0.426	1.99
MOLT-4	0.352 ± 0.031	2.2	1.28	2.512
RPMI-8226	0.394 ± 0.041	1.93	0.468	1.99
SR	0.284	2.09	0.386	1.26
**Non-Small Cell Lung Cancer**				
HOP-92	0.651 ± 0.044	1.79	1.13	3.16
**Colon Cancer**				
COLO 205	0.315 ± 0.038	1.79	1.37	7.94
HCT-116	0.299 ± 0.013	1.53	1.04	7.94
HT29	0.589 ± 0.12	2.00	1.61	5.01
SW-620	0.413 ± 0.061	1.73	0.736	3.98
**Melanoma**				
LOX IMVI	0.75 ± 0.33	1.67	0.298	1.99
MALME-3M	0.176 ± 0.02	0.33	1.32	1.99
M14	0.367 ± 0.05	1.50	1.50	3.16
MDA-MB-435	0.327 ± 0.014	1.61	1.35	7.94
SK-MEL-28	0.309 ± 0.102	1.50	1.19	3.98
SK-MEL-5	0.195 ± 0.004	1.07	1.80	2.51
UACC-257	0.769 ± 0.331	1.39	0.692	3.98
UACC-62	0.763 ± 0.277	1.80	1.58	3.16
**Ovarian Cancer**				
IGROV1	0.825 ± 0.095	1.90	1.51	1.99
OVCAR-3	0.252 ± 0.012	1.83	1.15	2.51
OVCAR-4	0.667 ± 0.16	1.32	1.54	5.01
OVCAR-8	0.873 ± 0.397	1.90	1.04	3.16
SK-OV-3	1.64 ± 0.15	6.41	18.9	10.0
**Renal Cancer**				
RXF 393	0.31 ± 0.027	2.15	1.42	3.16
**Prostate Cancer**				
PC-3	0.979 ± 0.181	2.03	2.41	3.98
DU-145	0.795 ± 0.04	1.81	2.15	7.94
**Breast Cancer**				
MCF7	0.367 ± 0.066	1.35	0.653	2.51
MDA-MB-231/ATCC	1.57 ± 0.02	1.94	1.17	3.98
BT-549	0.343 ± 0.014	1.54	1.25	1.25
T47D	0.355 ± 0.093	1.55	1.30	100
MDA-MB-468	0.283 ± 0.079	1.59	1.07	—
**MGM**	1.17	1.75	1.81	3.77

GI_50_values, defined as the concentration that inhibits growth by 50% from NCI screening. Mean graph medium (MGM) as average GI_50_ (µM) over all cell lines investigated.

In terms of potential candidates for the treatment of malignant diseases, the results from these studies are highly significant. Furthermore, the ability of these compounds to be used in reduced amounts in comparison to toxic ATO is attractive. The present study encourages further evaluation of the molecular mechanism of these peculiar organoarsenicals, in particular compound **2**, in inducing cell inhibition of solid tumors. This investigation may be interesting also in relation to the recent evidence where As_4_O_6_ has been successfully applied on human colon cancer, suppressing the NF-κB and NF-κB regulated genes involved in anti-apoptosis, proliferation, invasion and angiogenesis, with a mechanism of anticancer activity still remaining not completely understood^23^.

## Conclusion

In this study Arsenicin A analogues were obtained by a microwave assisted reaction using arsenic(III) trioxide and the proper pair of carboxylic acid/anhydride. The products include members of methylene homologue series, besides the racemic natural metabolite. Both IR and NMR data obtained for compound **5**, here synthesized for the first time, was in good agreement with the results obtained by the previous simulated spectroscopic analyses. This evidence strongly supports the role of computational approach in the structural elucidation of this class of arsenicals. The simple procedure here reported provides a novel route for the fast and efficient synthesis of organic tetraarsenicals, easily HPLC purified to be used for their bioactivity evaluation. Furthermore the method reduces the risk associated with the manipulation of these dangerous chemicals. The reaction can easily be scaled up to obtain the higher quantities required for *in vivo* studies. It can be associated to the column liquid chromatography of the crude mixture from MW-assisted reaction to provide pure compounds, taking advantage of UV detectable spots on TLC of the eluted fractions.

Based on arsenicin A as hit molecule, the obtained products were evaluated in the NCI -*in vitro* cytotoxicity screening on a full panel including leukemia and solid human cancer cell lines. Each tested arsenical resulted in being more active than arsenic trioxide, a FDA-approved drug in the treatment of leukemia, with the most lipophilic product **2** emerging as the molecule displaying the highest values of growth inhibition. These results warrant further biological studies, which can also include analogues containing sulfur atoms, based on the known affinity of arsenic for this element. Their structural feature could improve the lipophilic properties suitable for a drug development. In summary, the novel synthetic procedure to Arsenicin A and its analogues associated to the *in vitro* cytotoxicity evaluation described in this report may open promising perspectives in the development of new more potent and selective arsenical agents in cancer therapy.

## Materials and Methods

### Chemistry

The reagents and solvents were used without purification. All evaporations were carried out at room temperature at reduced pressure. CAUTION: due to the hazard statement for arsenic trioxide (H350), a reduced reaction scale and all the safety conditions in workup and manipulation were adopted. The reaction yields were calculated on product **2** or on a mixture of products (**3**, **4**, **5** and **1**) after chromatographic purification. Microwave (MW)-assisted reactions were carried out in sealed reaction vessels by using a mono-mode CEM Discover SP reactor. Thin layer chromatography (TLC) was carried out on Merck Kieselgel 60 PF_254_ and reversed phase Merck RP-18 F_254_, with visualization by either UV light or by treatment with an acid solution of cerium sulfate. Flash-chromatography (FC) was achieved on Merck silica gel 60 (15 ÷ 25 μm); preparative thin layer chromatography (PLC) on 20 × 20 cm Merck Kieselgel 60 F254 0.5 mm plates. HPLC purification was performed by a Merck Hitachi L-6200 apparatus, equipped with a diode array detector Jasco UVIDEC 100Vand a LiChrospher CN column, in isocratic conditions with eluent hexane/AcOEt 96:4, flow 5 mL·min^−1^, detection at 254 nm. Infrared spectra were recorded by using a FT-IR Tensor 27 Bruker spectrometer equipped with Attenuated Transmitter Reflection (ATR) device at 1 cm^−1^ resolution in the absorption region Δν 4,000 ÷ 1,000 cm^−1^. A thin solid layer was obtained by the evaporation of the methanol solution in the sample. The instrument was purged with a constant dry nitrogen flow. Spectra processing was made using Opus software packaging. NMR spectra were recorded on a Bruker-Avance 400 spectrometer by using a 5 mm BBI probe ^1^H at 400 MHz and ^13^C at 100 MHz in CDCl_3_ (by previous treatment on basic alumina to avoid acidic traces), relative to the solvent residual signals δ_H_ = 7.25 and δ_C_ = 77.00 ppm, *J* values in Hz. Structural assignments are from ^1^H, ^1^H-COSY, heteronuclear single quantum correlation (HSQC) and heteronuclear multiple bond correlation (HMBC) experiments. APCI-MS and tandem (MS/MS)^n^ were taken with a Bruker Esquire-LC mass spectrometer equipped with an atmospheric pressure chemical ionization ion (APCI) source used in positive ion mode. The sample was injected into the source from a methanolic solution. Electron ionization (EI)MS (*m/z*, rel.%) and high resolution (HR)-EIMS spectra were taken with a Kratos-MS80 mass spectrometer equipped with a home-built computerized acquisition system.

### Microwave-assisted synthesis, purification and structural characterization of 9,10-dimethyl-2,4,6,8-tetraoxa-1,3,5,7,-tetraarsa-tricyclo[3.3.1.13,7]decane (2)

Propionic acid (0.32 mL, 0.44 mmol), propionic anhydride (1.15 mL, 0.90 mmol) and anhydrous K_2_CO_3_ (15.6 mg, 0.11 mmol) were added to arsenic (III) oxide (22.1 mg, 0.11 mmol) and the mixture subjected to microwave irradiation in a CEM apparatus at 160 °C for 20 min. After the addition of H_2_O (0.05 mL), the reaction mixture was MW-irradiated at 80 °C for additional 20 min. The reaction progress was monitored by TLC (hexane-AcOEt 9:1), observing the appearance of a spot at R_f_ = 0.9. The product was extracted by using CH_2_Cl_2_ (4 mL × 4). The combined organic phases were dried by the addition of anhydrous sodium sulfate, filtered and evaporated at reduced pressure at room temperature. The residue was purified by preparative TLC using 1:1 dichloromethane/hexane as the eluent. The product was further purified by quantitative HPLC analysis (LiChrospher CN column, hexane 100%, 5 mL·min^−1^, λ 240 nm), to obtain pure **2** as a whitish powder by evaporating fraction eluted at t_R_ 6.4 min (19.2 mg, 82%). The two products corresponding to very minor peaks with longer retention times were not considered. Compound **2** gave superimposable data to the ones obtained for the product we previously synthesized by the conventional heating procedure^10^.

### Microwave assisted synthesis of products 1 and 3–5

After suitable changes in the experimental conditions involving temperature values, reaction times and molar ratio of the reagents, the following procedure was selected. Acetic acid (0.85 mL, 1.47 mmol), acetic anhydride (0.28 mL, 2.94 mmol) and anhydrous K_2_CO_3_ (50.85 mg, 0.368 mmol) were added to arsenic (III) oxide (68 mg, 0.368 mmol) and the mixture was subjected to microwave irradiation in a CEM apparatus at 138 °C for 30 min. After addition of H_2_O (0.15 mL) the reaction mixture was MW-irradiated at 80 °C for additional 30 min. The reaction progress was monitored by TLC (hexane- AcOEt 9:1), observing the appearance of a spot at R_f_ = 0.85. Additional water (3 mL) was added and the mixture was extracted with CH_2_Cl_2_ (4 mL × 4). The combined organic phases were dried by the addition of anhydrous sodium sulfate, filtered and evaporated at reduced pressure at room temperature. The residue (mg 45.9, 64% global yield) was purified by PLC using dichloromethane 1:1 as the eluent.

### Synthesis of arsenicals 3–5 by conventional heating

A mixture of acetic acid (1.7 mL, 2.94 mmol), acetic anhydride (0.56 mL, 5.88 mmol) anhydrous K_2_CO_3_ (102.3 mg, 0.74 mmol) and arsenic (III) oxide (146.5 mg, 0.74 mmol) was heated at 140 °C whilst stirring for 3 h. After the addition of H_2_O (0.3 mL), heating continued for an additional 2 hours. The same workup previously reported for MW-assisted procedure has been carried out, obtaining a residue (54.8 mg, 38%).

### HPLC purification of the products

The product mixture recovered by preparative TLC showed the presence of different components by NMR and MS analysis. Pure products were obtained by subjecting it to a preparative HPLC analysis (LiChrospher CN column, hexane/AcOEt 96:4, flow 5 mL·min^−1^, 254 nm). Starting from the residue obtained by MW-assisted synthesis, pure arsenicals were obtained by evaporating the fractions eluted for peaks at t_R_ = 6.2 min (**3**, mg 30.4), t_R_ = 7.4 min (**4**, mg 3.0), t_R_ = 8.7 min ((±)-arsenicin A, **1**, mg 0.9) and t_R_ = 11.0 min (**5**, mg 8.9).

### Comparison between the synthetic procedures

By using the same molar ratio of reagents, oil bath heating (140 °C, 5 h) and MW irradiation (138 °C, 1 h) conditions were compared for the production of compounds **3–5** and (±)-**1**, observing in the latter case a higher global yield. After HPLC purification and assignment of NMR values for each arsenical, their relative amount was deduced by integrating signals in^1^ HNMR spectra of the two crude reaction mixtures (**3**: 1.85 s, 2 H; **4**: 2.71, d, 2 H; **1**: 2.42 d, 2 H; **5**: 3.11, d, 2 H). It was established that products **3**, **4**, (±)-**1** and **5** were obtained in 82:10:3:5 ratio by MW irradiation and in 71:7:1:21 ratio by conventional heating, respectively.

### 2,4,6,8-Tetraoxa-1,3,5,7-tetraarsaadamantane (3)

Whitish powder. UV (MeOH) λ_max_ (ε) 230 (7740), 276 (6220) nm. FT-IR (cm^−1^) 1076, 808, 713 cm^−1^, assignments in Supplementary Table S1. ^1^HNMR (400 MHz, CDCl_3_) δ_H_ 1.85 (s). ^13^CNMR δ_C_ 30.60. APCI(+)MS: *m/z* 393[M + H]^+^, APCI(+)MS/MS (393): *m/z* 375, 363, 267; EIMS (70 eV) *m/z* (%): 393 ([M + 1]^+^, 2), 392 (M^+•^, 100), 362 (M^+•^- H_2_CO, 98); HR-EIMS: *m/z* 391.6972 ± 0.0020, calculated for M^+•^ C_2_H_4_As_4_O_4_ 391.6973, *m/z* 361.6865 ± 0.0020, calculated for [M^+•^- H_2_CO] CH_2_As_4_O_3_ 361.6867.

### 2,4,6,9-Tetraoxa-1,3,5,7-tetraarsaadamantane (4)

Whitish powder. FT-IR (cm^−1^) 1103, 796, 754, 681 cm^−1^, assignments in Supplementary Table S1. ^1^HNMR (400 MHz, CDCl_3_) δ_H_ 2.71(d, *J*_gem_ 13.8 Hz, 2 H), 1.71(*d*, *J*_gem_ 13.8 Hz, 2 H). ^13^CNMR (100 MHz, CDCl_3_) δ_C_ 23.71. APCI(+)MS: *m/z* 393[M + H]^+^, APCI (+)MS/MS (393): *m/z* 375, 363, 267; EIMS (70 eV) *m/z* (%) 392 (M^+•^,100), 362 (M^+•^- H_2_CO, 95); HR-EIMS *m/z* 391.6969 ± 0.0020, calculated for M^+•^ C_2_H_4_As_4_O_4_ 391.6973.

### 2,4,10-Trioxa-1,3,5,7-tetraarsaadamantane (5)

Whitish powder. UV (MeOH) λ_max_ (ε) 229 (7490), 284 (3210) nm. FT-IR (cm^−1^) 1118, 786, 684, assignments in Supplementary Table S1. ^1^HNMR (400 MHz, CDCl_3_) δ_H_ 3.11(d, *J*_gem_ 13.8 Hz, 3 H), 1.53 (*d*, *J*_gem_ 13.8 Hz, 3 H). ^13^CNMR (100 MHz, CDCl_3_) δ_C_ 17.82 ppm. APCI(+)MS: *m/z* 391 [M + H]^+^, APCI (+)MS/MS (391): *m/z* 375, 361, 253, 225; EIMS (70 eV): *m/z* (%) 390 (M^+•^,100), 360 (M^+•^-H_2_CO, 98); HR-EIMS: *m/z* 389.7175 ± 0.0020, calculated for M^+•^ C_3_H_6_As_4_O_3_ 389.7181; *m/z* 359.70748 ± 0.0020, calculated for [M^+•^-H_2_CO] C_2_H_4_As_4_O_2_ 359.70751.

### Computational details for simulated IR spectra

Quantum chemical calculations were performed on a Pentium IV/3.6 GHz personal computer using the Gaussian 03 W revision E.01 package program set^24^. The basis set of choice was 6–311 G(d,p) for geometry optimization and the optimized structural parameters were employed in the vibrational energy calculations at the DFT levels to characterize all stationary points as minima. Then, vibrational averaged nuclear positions were adopted for harmonic vibrational energy calculations, resulting in IR wavenumbers together with intensities and force constants. For each optimized structure, no imaginary wavenumber modes were obtained, proving that a local minimum on the potential energy surface was found. The gradient-corrected DFT with the three-parameter hybrid functional (B3)^25^ for the exchange part and the Lee–Yang–Parr (LYP) correlation function^26^ were utilized.

### *In vitro* anticancer screening

Compounds **2**, **4** and **5** were evaluated for their *in vitro* activity against cancer cell lines by the National Cancer Institute (NCI) following its anticancer drug development programme based on automated sulforhodamine blue (SRB) cytotoxicity assay. The screening is a two-stage process, where after a first evaluation carried out against the full panel of cell lines at a single dose of 10 µM, the compounds exhibiting significant growth inhibition are tested at five concentration levels^22^.

## Electronic supplementary material

Supplementary Information
