# Supplementary material for: Synthesis and in-vitro anticancer evaluation of polyarsenicals related to the marine sponge derived Arsenicin A
Source: Sci Rep. 2017 Sep 14;7:11548. doi: 10.1038/s41598-017-11566-6 (PMC5599660; doi:10.1038/s41598-017-11566-6)
Supplement: Supplementary file 1 — Supplementary Information [file 41598_2017_11566_MOESM1_ESM.pdf]

Supplementary Information

# Synthesis and *in-vitro* anticancer evaluation of polyarsenicals related to the marine sponge derived Arsenicin A

Ines Mancini,* Matteo Planchestainer and Andrea Defant

*Laboratorio di Chimica Bioorganica, Dipartimento di Fisica, Univesità di Trento , via Sommarive 14, I-8123 Povo-Trento, Italy*

E-Mail: ines.mancini@unitn.it

## Figures and Table

**Figure S1.** Electron ionization mass spectrum (EIMS) of compound **3** ($C_2H_4As_4O_4$, *m/z* 391.7).

**Figure S2.** EIMS spectrum of compound **4** ($C_2H_4As_4O_4$, *m/z* 391.7).

**Figure S3.** EIMS spectrum of compound **5** ($C_3H_6As_4O_3$, *m/z* 389.7).

**Figure S4.** $^1$HNMR spectrum (top, 1.58 ppm signal for $H_2O$) and $^1$H,$^{13}$C correlation by HSQC experiment ( down) of compound **3** (400 MHz, $CDCl_3$).

**Figure S5**. $^{13}$CNMR spectrum of compound **3** (100 MHz, $CDCl_3$).

**Figure S6.** $^1$HNMR spectrum (top, 1.58 ppm signal for $H_2O$) and $^1$H,$^{13}$C correlation by HSQC experiment (down) of compound **4** (400 MHz, $CDCl_3$).

**Figure S7**. $^{13}$CNMR spectrum of compound **4** (100 MHz, $CDCl_3$).

**Figure S8.** $^1$HNMR spectrum (top, 1.58 ppm signal for $H_2O$) and $^1$H,$^{13}$C correlation by HSQC experiment (down) of compound **5** (400 MHz, $CDCl_3$).

**Figure S9**. $^{13}$CNMR spectrum of compound **5** (100 MHz, $CDCl_3$).

**Figure S10.** Experimental FT-ATR (top) and DFT-calculated (bottom) IR spectra of compound **3**. Comparison of wavenumber values in Table S1.

**Figure S11.** Experimental FT-ATR (top) and DFT-calculated (bottom) IR spectra of compound **4**. Comparison of wavenumber values in Table S1.

**Figure S12.** FTATR- IR spectrum of compound **5**. Comparison with wavenumber values by DFT-simulated spectrum is reported in Table S1.

**Table S1** Experimental IR data and calculated frequencies from density functional theory (DFT)-vibrational analysis for compounds **3-5** and their energy- minimized structures.

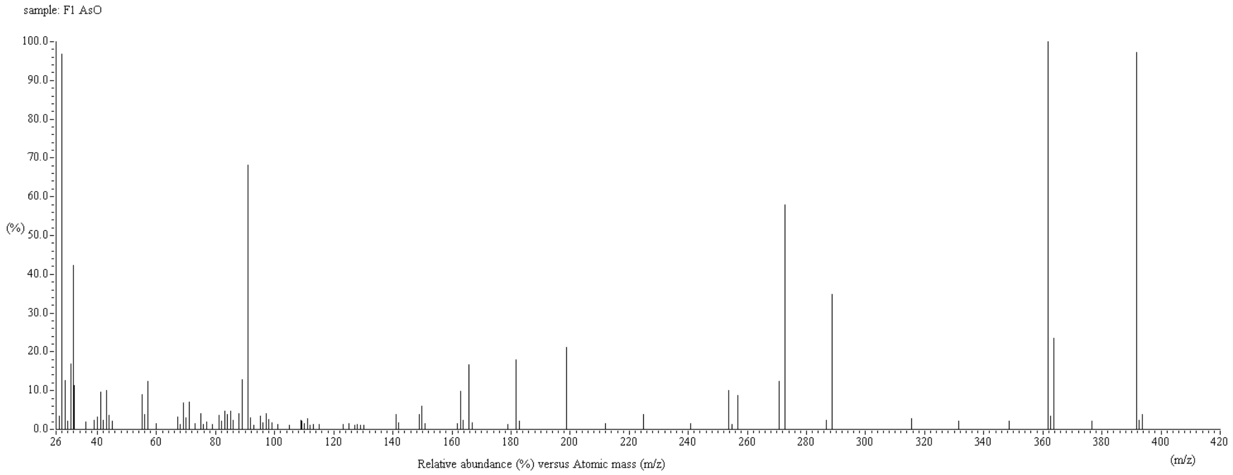


**Figure S1**. Electron ionization mass spectrum (EIMS) of compound **3** ($C_2H_4As_4O_4$, *m/z* 391.7).

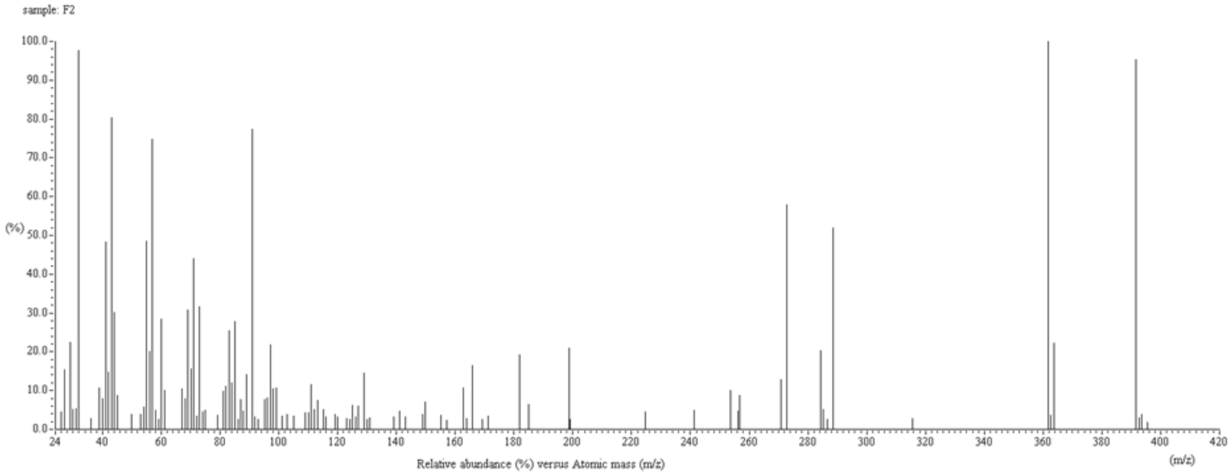


**Figure S2.** EIMS spectrum of compound **4** ($C_2H_4As_4O_4$, *m/z* 391.7).

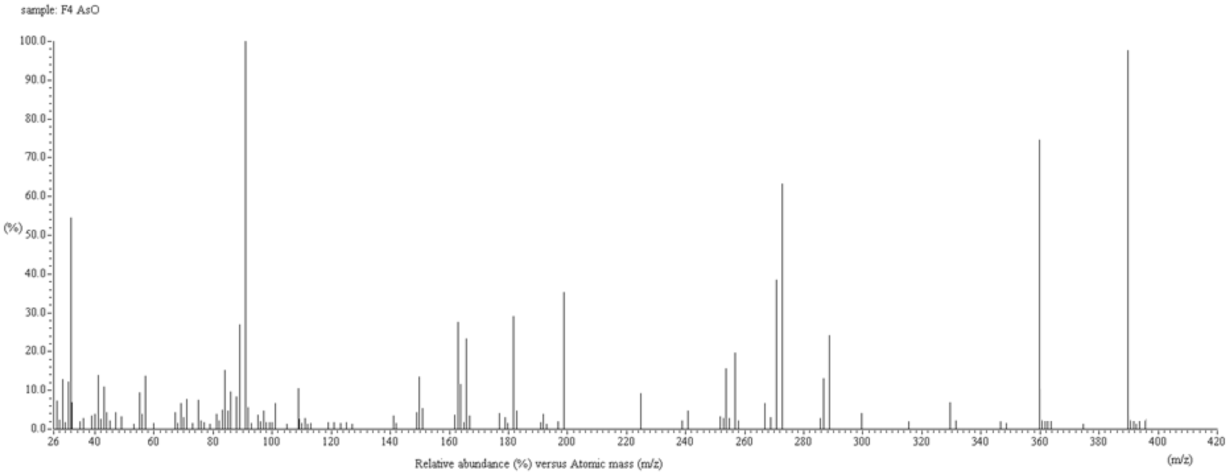


**Figure S3.** EIMS spectrum of compound **5** ($C_3H_6As_4O_3$, *m/z* 389.7).

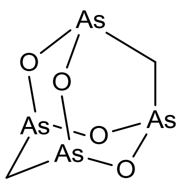


**3**

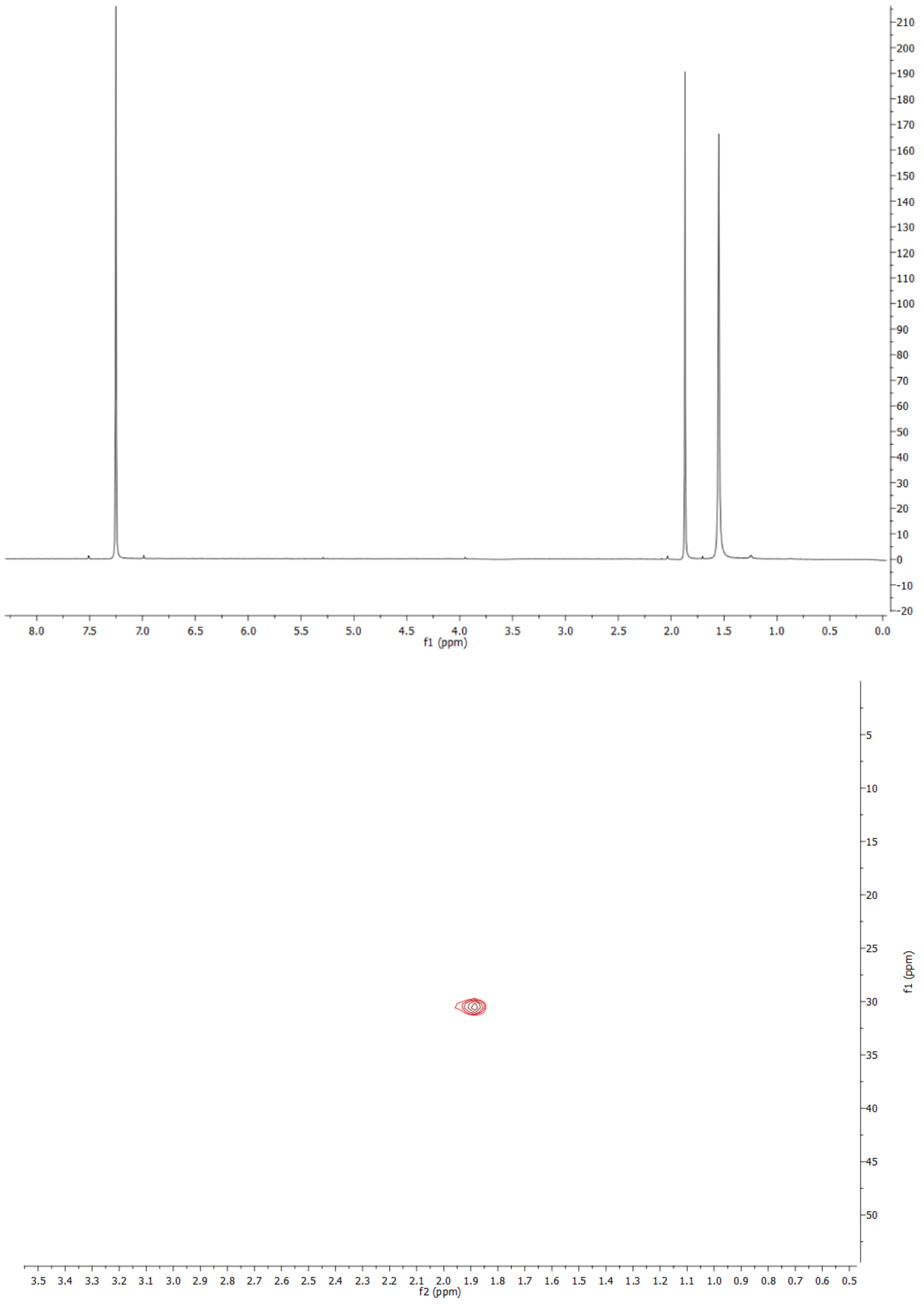


**Figure S4.** $^{1}$HNMR spectrum (top, 1.58 ppm signal for $H_2O$) and $^{1}$H,$^{13}$C correlation by HSQC experiment (down) of compound **3** (400 MHz, $CDCl_3$)

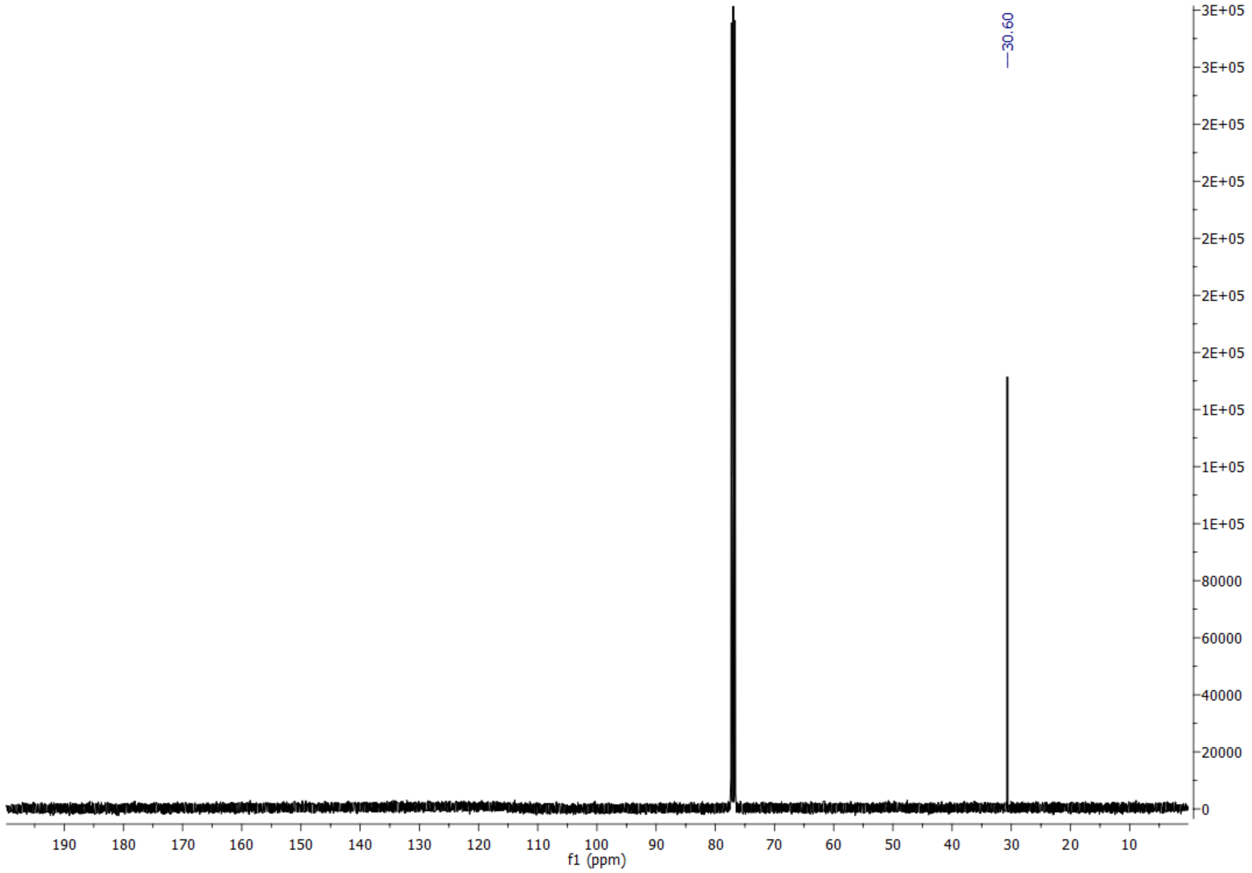


**Figure S5**. $^{13}$CNMR spectrum of compound **3** (100 MHz, $CDCl_3$).

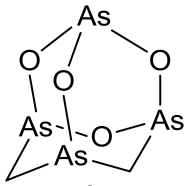


**4**

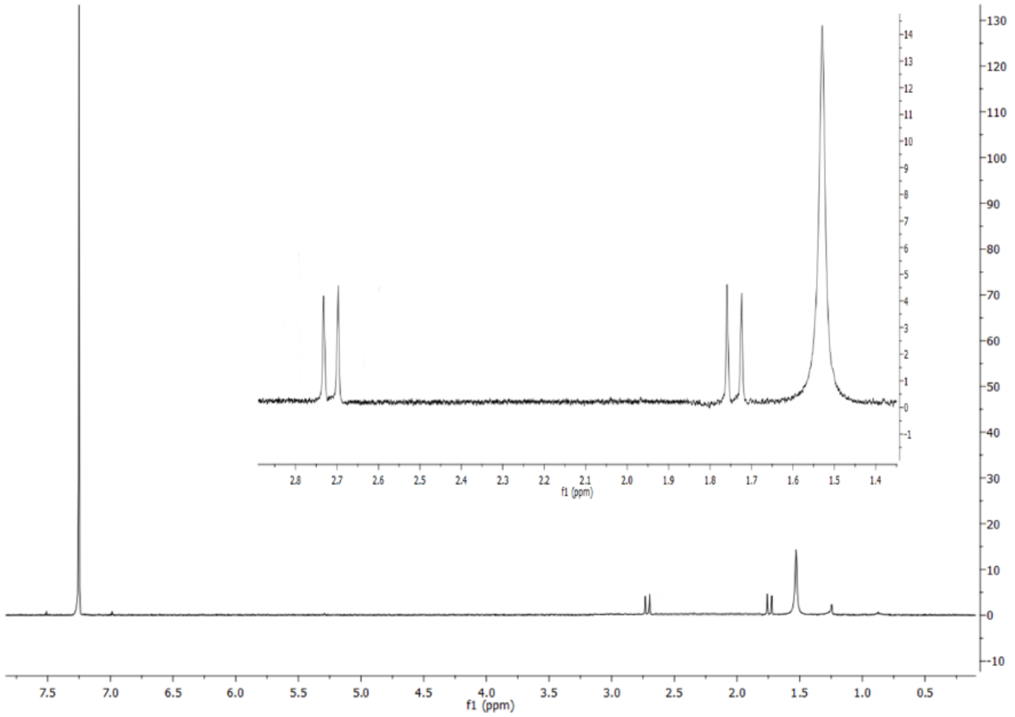


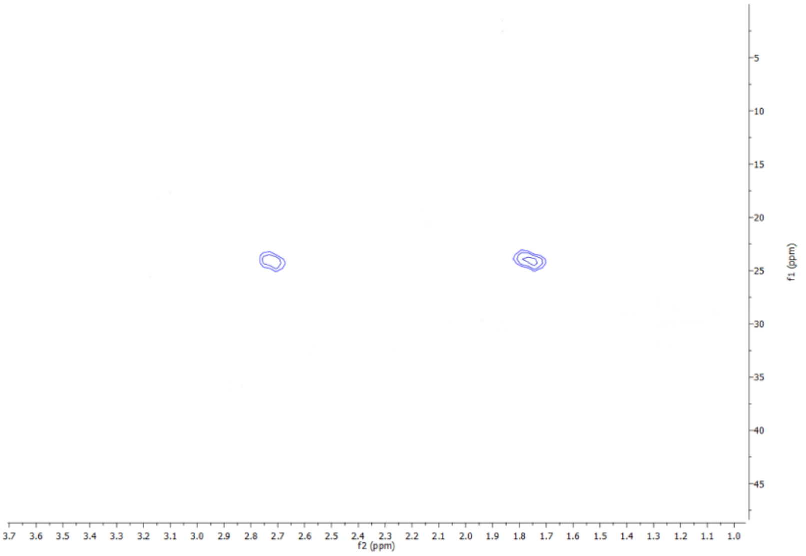


**Figure S6.** $^{1}$HNMR spectrum (top, 1.58 ppm signal for $H_2O$) and $^{1}$H,$^{13}$C correlation by HSQC experiment (down) of compound **4** (400 MHz, $CDCl_3$).

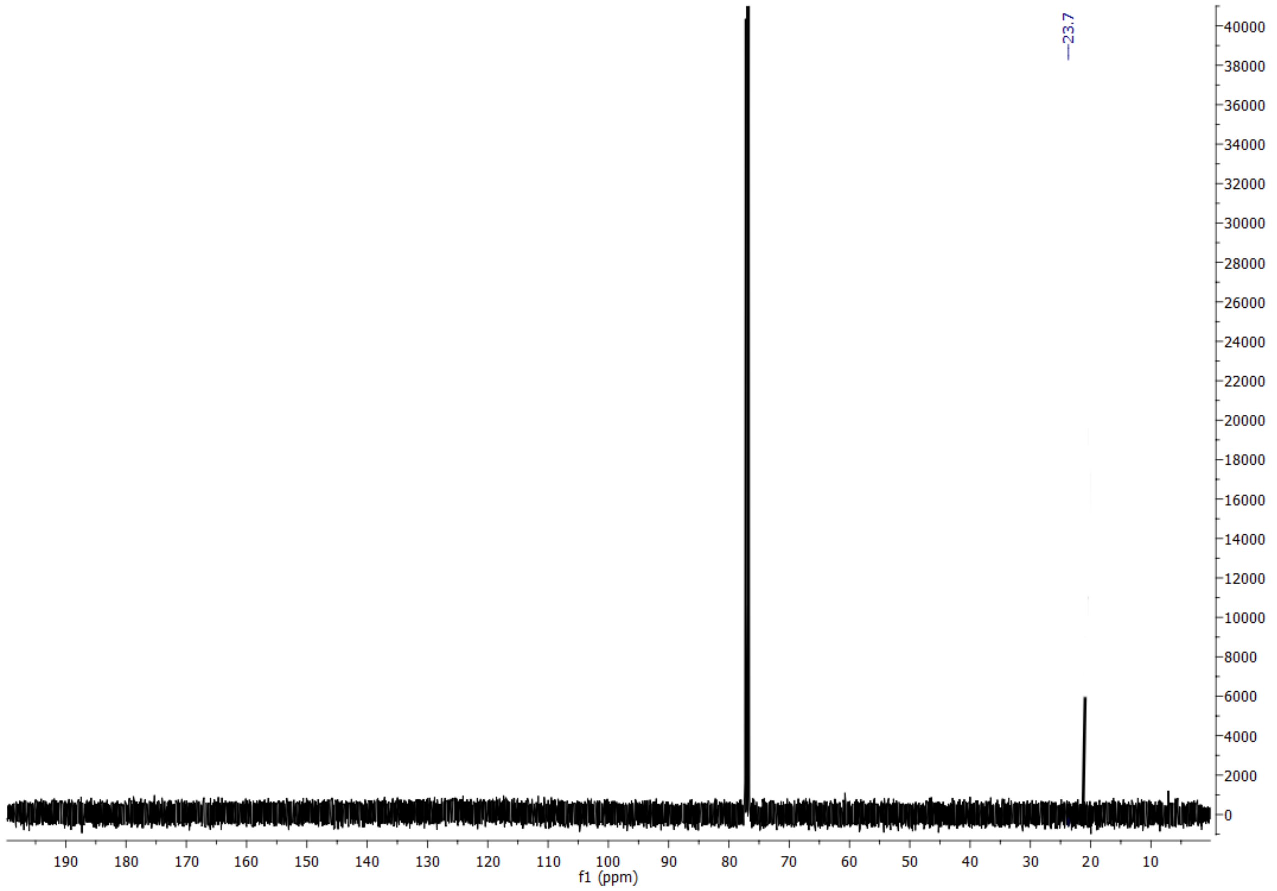


**Figure S7**. $^{13}$CNMR spectrum  of compound **4** (100 MHz, $CDCl_3$).

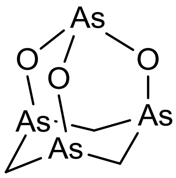

**5**

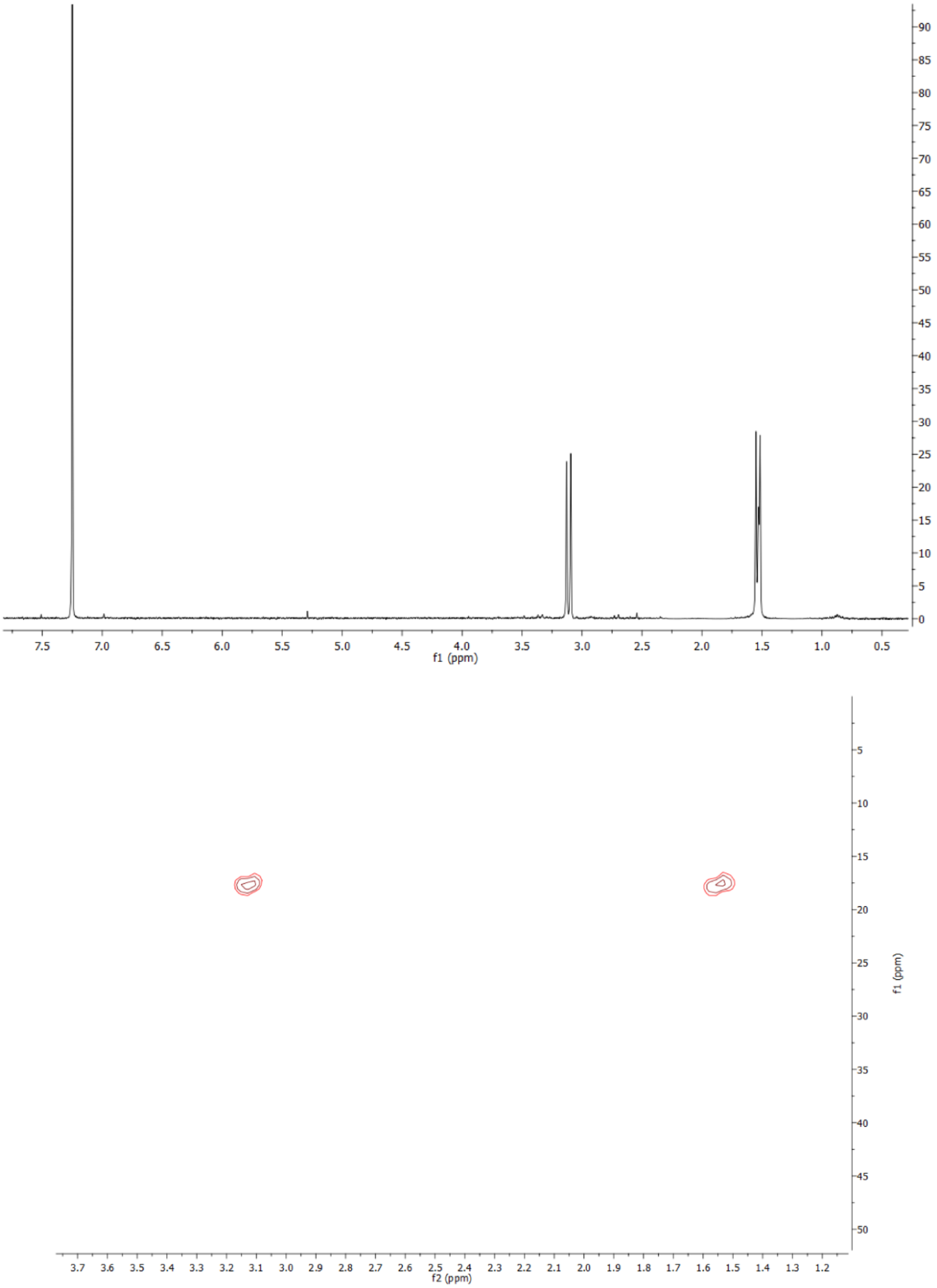


**Figure S8.** $^{1}$HNMR spectrum (top, 1.58 ppm signal for $H_2O$) and $^{1}$H,$^{13}$C correlation by HSQC experiment (down) of compound **5** (400 MHz, $CDCl_3$).

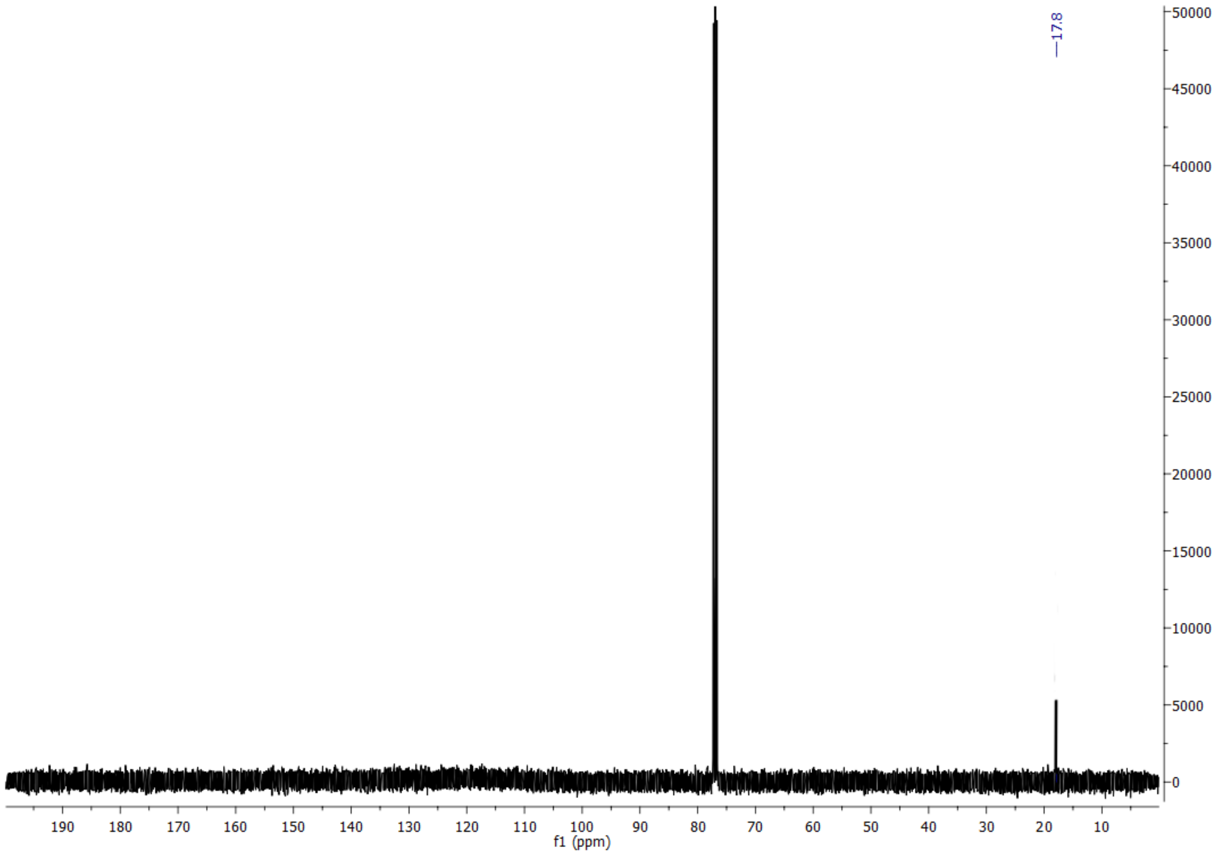


**Figure S9**. $^{13}$CNMR spectrum of compound **5** (100 MHz, $CDCl_3$).

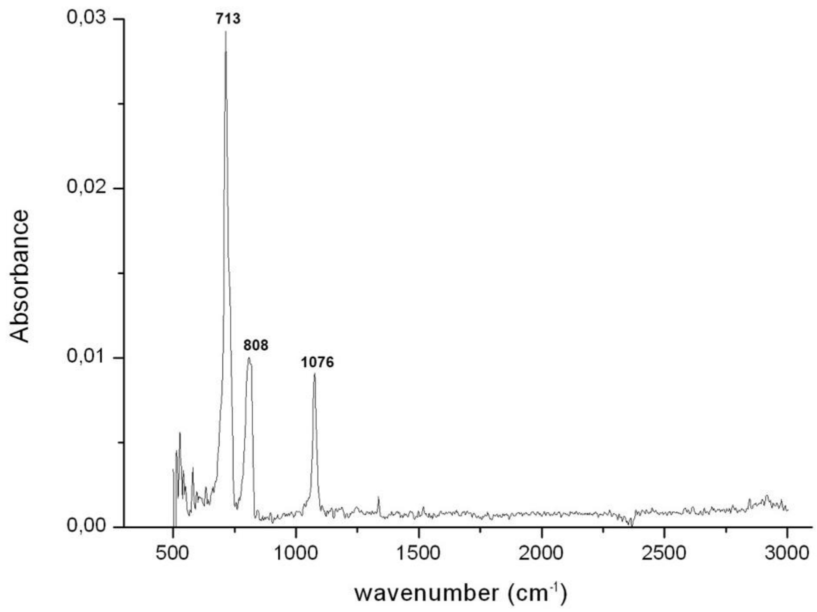


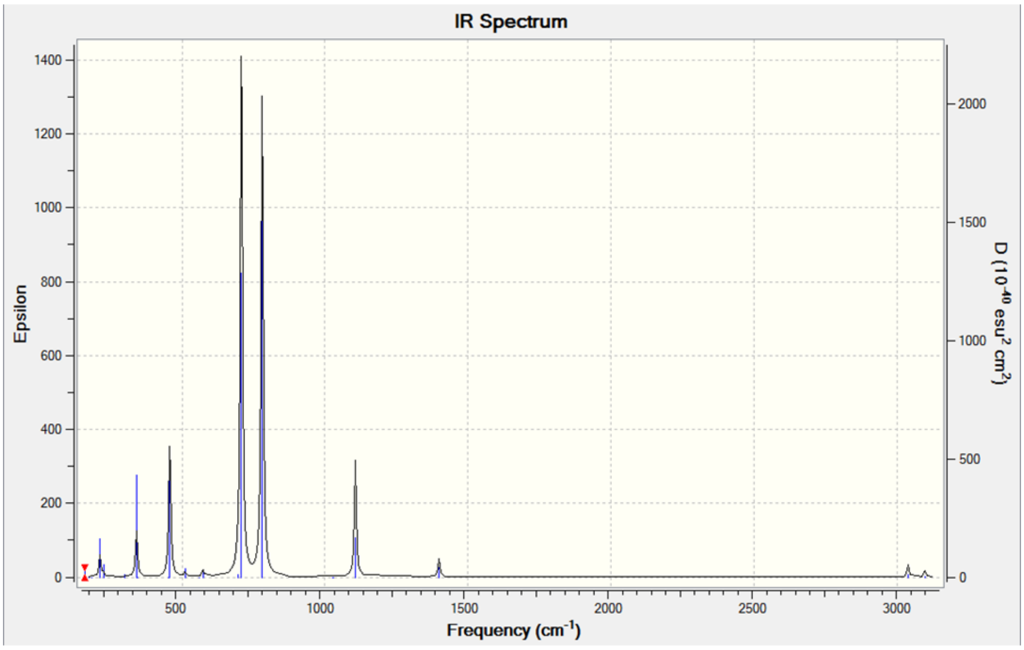


**Figure S10.** Experimental FT-ATR (top) and DFT-calculated (bottom) IR spectra of compound **3**. Comparison of wavenumber values in Table S1.

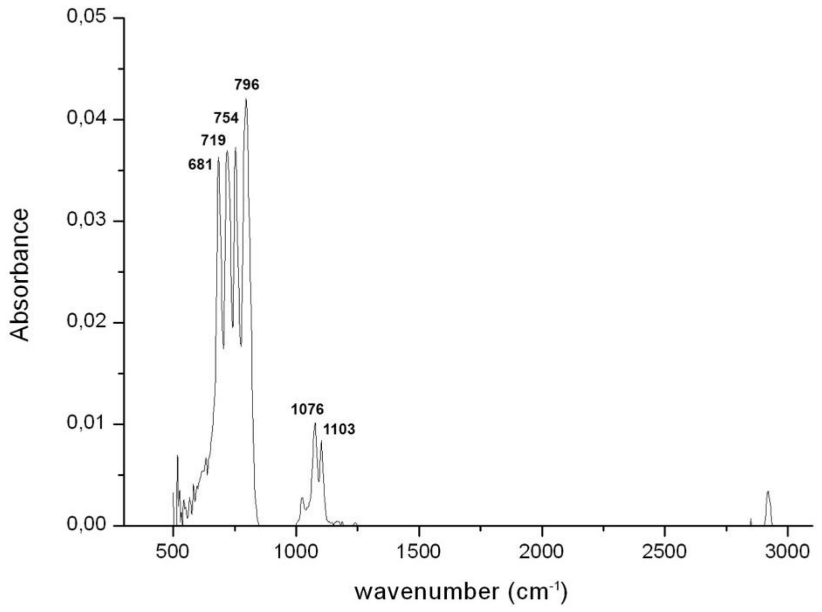


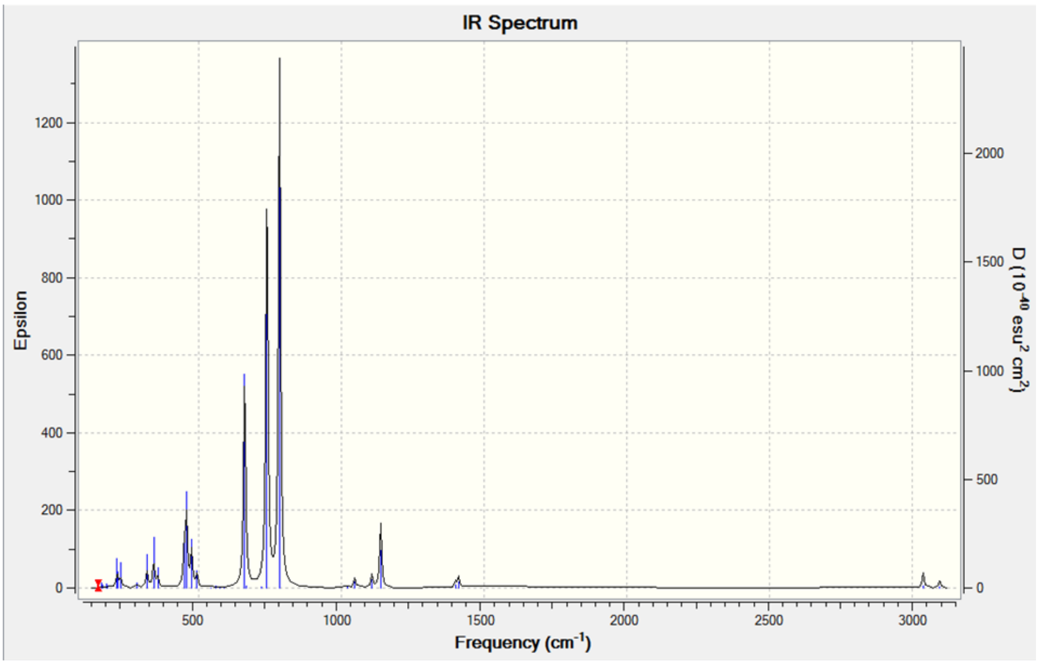


**Figure S11.** Experimental FT-ATR (top) and DFT-calculated (bottom) IR spectra of compound **4**. Comparison of wavenumber values in Table S1.

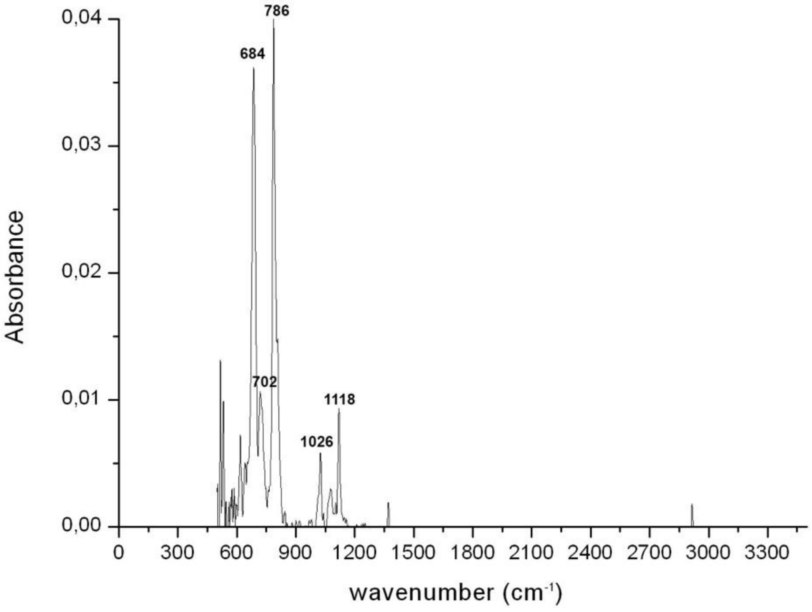


**Figure S11.** FTATR- IR spectrum of compound **5**.

**Table S1.** Experimental IR data and calculated frequencies from DFT-vibrational analysis for compounds **3-5** and their energy- minimized structures.

| IR frequency ($cm^{-1}$) Calculated [a] | Experim. | Relative Intensity | Assignment | Minimized Structure |
|---|---|---|---|---|
| | | | *Compound* **3** | |
| 708<br>780<br>1106 | 713<br>808<br>1076 | v strong<br>strong<br>weak | As-C stretching<br>As-O stretching<br>C-H bending | 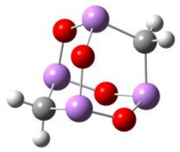 |
| | | | *Compound* **4** | |
| 678<br>760<br>805<br>1156 | 681<br>754<br>796<br>1103 | v strong<br>v strong<br>v strong<br>weak | ring deformation<br>As-C stretching<br>As-O stretching<br>C-H bending | 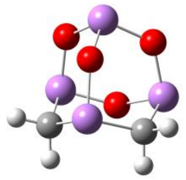 |
| | | | *Compound* **5** | |
| 670<br>777<br>1162 | 684<br>786<br>1118 | v strong<br>v strong<br>weak | ring deformation<br>As-O stretching<br>C-H bending | 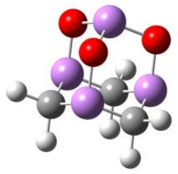 |

[a] Calculated values for **5**, reported by Mancini et al. *Chem. Eur. J.* **2006**, *12*, 8989.
